# Differences in the percutaneous nephrolithotomy practice patterns among Latin American urologists with and without endourology training

**DOI:** 10.1590/S1677-5538.IBJU.2017.0599

**Published:** 2018

**Authors:** Braulio O. Manzo, Edgar Lozada, Fabio C. Vicentini, Francisco Javier Sanchez, Gildardo Manzo

**Affiliations:** 1 Hospital Regional de Alta Especialidade do Bajío, México; 2 Hospital das Clínicas da universidade de São Paulo, São Paulo, SP, Brasil; 3 Centro de Urologia do México (UROCEM), México

**Keywords:** Nephrolithotomy, Percutaneous, Health Care Surveys, Kidney Calculi

## Abstract

**Purpose:**

Although the worldwide percutaneous nephrolithotomy (PCNL) practice pat- terns determined via a survey sent to members of the Endourological Society have been published, differences in PCNL practice patterns among Latin American urologists based on endourological or lithiasis training have not been published.

To determine the PCNL practice patterns among Latin American urologists with and without training in endourology.

**Materials and Methods:**

The SurveyMonkey® web platform was used to develop a 27-item survey on PCNL for the treatment of renal stones, and the survey was sent via e- -mail and other electronic media to 2000 urologists from 15 Latin American countries. Endourology-trained (group 1) and nontrained urologists (group 2) were analyzed. The group results were compared using the chi-squared and Fisher's exact tests. SPSS version 20 for Windows was used for all analyses.

**Results:**

A total of 331 urologists responded to the survey (rate of 16.55%): 221 (66.7%) in group 1 and 110 (33.2%) in group 2). In groups 1 and 2, 91.9% and 63.2% performed PCNL, respectively: 85.1% and 58.5% used preoperative tomography, respectively; 12.7% and 4.7% used preoperative nephrolitometry nomograms, respectively: 45.2% and 32.1% used endoscopic combined intrarenal surgery, respectively: 68.3% and 38.7% used mul- tiple percutaneous tract realization, respectively: and 19.9% and 5.7% used minimally invasive PCNL, respectively (all p=0.0005).

**Conclusions:**

Statistically significant differences were observed in PCNL practice patterns of Latin American urologists with and without training in endourology. Specific training in endourology significantly influence the practice patterns of Latin American urologists.

## INTRODUCTION

Currently, percutaneous nephrolithotomy (PCNL) is the gold standard treatment for renal stones >20mm and >15mm in diameter that are located in an inferior renal calyx (1, 2). In recent years, PCNL procedures have significantly increased worldwide. Although PCNL is an established procedure with specific indications, differences are observed in the practice patterns among urologists worldwide (3), which has contributed to significant differences in surgical procedures, preoperative planning, and postoperative management. These significant differences could impact the final surgical outcomes and may occur because of factors that include the urologist's training, experience and case volume and the practice setting. Because PCNL is a multiple step procedure, the likelihood of modifications by urologists at every single step is increased.

Until recently, few reports have been published on the particular practice patterns among urologists, and these studies were mainly conducted in the United States and Europe (3–5). Mo-reover, limited data are available on the practice patterns among Latin American urologists. Therefore, our aim was to evaluate the practice patterns among Latin American urologists and compare the impact of endourology training on the performance of PCNL. Endourology training is defined as a post-residency sub-specialization in all minimally invasive surgeries, including percutaneous surgery for urinary stones treatment. To our knowledge, this study is the first to report information on the PCNL practice patterns of urologists in relation to endourology training.

## MATERIALS AND METHODS

The web platform SurveyMonkey® was used to create a 27-item anonymous survey on the PCNL for renal stone treatment (see appendix). The survey was sent via e-mail and other electronic media to 2000 urologists from 15 different Latin American countries, and a Whatsa-pp group was created for communication among the main urologists in each country. The survey collected information on the demographics, age, nature of the practice, training in endourology (defined as a post-residency sub-specialization specific for urinary stones treatment) and pre-operative, perioperative and postoperative variables. The results were analyzed based on the following two groups: endourology-trained urologists (group 1) and nontrained urologists (group 2) that reported to do PCNL and we ex-cluded from the comparative analysis those urologists that do not perform PCNL. The group re-sults were compared using the chi-squared test and Fisher's exact test. The statistical program SPSS version 20 for Windows was used for all analyses. Statistical significance was defined at a p-value <0.05.

## RESULTS

### Demographic data

A total of 331 participants from 15 different countries responded to the survey (rate of 16.55%) Table-1. Of these, 257 of participants (77%) were under 50 years old and 74 >50 years old (23%). A total of 173 of the 257 participants under 50 years old (67.3%) reported having endourological training, whereas 48 (64.9%) of the 74 participants >50 years old reported endourological training, with no statistically significant difference between age group among trained and not trained urologists (p-value 0.403). Of the total number of participants, 221 (66.7%) respondents had endourology training, and 110 (33.2%) participants did not have endourology training.

**Table 1:** Participants by country.

Country	N	Percentage
Mexico	84	25.4%
Argentina	48	14.5%
Brazil	35	10.6%
Colombia	20	6%
Uruguay	18	5.4%
Peru	16	4.8%
Dominican Republic	11	3.3%
Ecuador	4	1.2%
Paraguay	15	4.5%
Venezuela	34	10.3%
Chile	12	3.6%
Nicaragua	15	4.5%
El Salvador	1	0.3%
Guatemala	9	2.7%
Panama	9	2.7%

For the subsequent analysis we excluded those urologists who do not perform PCNL and we created two groups for comparing those trained and not trained, as stated in our methods. In groups 1 and 2, 91.9% and 63.2% of participants performed PCNL (p=0.0005). Thus, the trained urologists performed significantly more PCNL procedures per year than the nontrained urologists (p=0.0005) (Table-2).

**Table 2:** Case volume per year for the trained and nontrained urologists.

Case volume/year	Trained urologists	Untrained urologists	p-value*
1-10	59 (26.7%)	27 (25.5%)	
11-30	63 (28.5%)	24 (22.6%)	
31-60	41 (18.6%)	10 (9.4%)	0.0005
61-100	23 (10.4%)	4 (3.8%)	
>100	15 (6.8%)	1 (0.9%)	

* Fisher's exact test was used. Differences were statistically significant at p-values <0.05.

### Preoperative considerations

With respect to the preoperative considerations, 72.9% and 51.9% of the urologists in groups 1 and 2 used renal stones >20mm as an indication for PCNL (p=0.0005), respectively; 27.1% and 11.3% of the urologists in groups 1 and 2 used renal stones >15mm in the lower pole calyx as an indicator for PCNL (p=0.003), respectively; 85.1% and 58.5% of the urologists in groups 1 and 2 considered preoperative tomography necessary (p=0.0005), respectively; and 12.7% and 4.7% of the urologists in groups 1 and 2 used preoperative nephrolithometry scores as an indicator (p=0.0005).

### Perioperative variables

Of the urologists with and without endourology training, a total of 45.2% and 32.1% used the practice pattern of endoscopic combined intrarenal surgery (ECIRS) (p=0.0005), respectively; 68.3% and 38.7% used multiple percutaneous tract realization of each group (p=0.0005), respec-tively; and 19.9% and 5.7% used minimally invasive percutaneous nephrolithotomy (MiniPERC) (p=0.0005), respectively (Table-3). Only 7 participants (2.1%) of the total reported performing percutaneous puncture guided by ultrasound.

**Table 3:** Comparison of variables between urologists with and without endourology training.

Fluoroscopic puncture technique		Trained	Untrained	p-value*
Bull's eyes		59 (29.5%)	19 (28.3%)	
0-90 degrees		67 (33%)	14 (20.9%)	0.01
Triangulation technique		64 (31.5%)	31 (46.2%)	
Other		13 (6.4%)	3 (4.5%)	
**Preferred position**				
	Prone	124 (61.1%)	48 (71.7%)	
	Supine (Valdivia)	19 (8.6%)	5 (7.5%)	0.01
	Supine (Valdivia-Galdakao)	50 (24.6%)	11 (16.4%)	
	Other	10 (4.9%)	3 (4.5%)	
**Dilation method**				
	Amplatz	74 (33.5%)	67 (63.2%)	0.005
	Alken	108 (48.9%)	24 (22.6%)	0.0001
	Baloom	13 (5.9%)	5 (4.7%)	0.6654
	One shot	16 (7.2%)	8 (7.5%)	0.9205
	Other	10 (4.5%)	2 (1.9%)	0.2350
**Preferred method for postopera-tive stone status**				
	Tomography	123 (55.7%)	79 (74.5%)	0.0010
	USG	14 (6.3%)	2 (1.9%)	0.0809
	Radiography	36 (16.3%)	15 (14.2%)	0.6178
	Radiography & USG	37 (16.7%)	10 (9.4%)	0.0779
	Other	11 (5%)	0 (0%)	0.0195
**Catheters after procedure**				
	Nephrostomy only	138 (62.4%)	84 (79.2%)	0.0023
	Nephrostomy & catheter	60 (27.11%)	20 (18.9%)	0.130
	Catheter only	7 (3.2%)	0 (0%)	0.0640
	Complete tubeless	6 (2.7%)	0 (0%)	0.0869
	Other	10 (4.5%)	2 (1.9%)	0.2350

* Fisher's exact test was used. Differences were statistically significant at p-values <0.05.

### Postoperative conduct

At the end of the procedure, the nephrostomy tube was left in place more frequently by the untrained urologists than by the endourology-trained urologists, and the difference was statistically significant (p=0.0023). Tomography was more frequently used as the stone-free evaluation method by untrained urologists (Table-3).

## DISCUSSION

PCNL is a complex minimally invasive procedure for renal stone treatment, and reports have shown that the outcomes are dependent on the case volume and experience of the surgeons. Kadlec showed that the in-hospital mortality rates were lower at higher-volume centers (6), and Withington demonstrated that the length of the hospital stay was shorter in higher-volume units (7, 8). These results may be related to the different practice patterns of urologists dedicated predominantly to stone disease and urologists who are more generalized. Moreover, practice patterns vary among urologists worldwide (5). Our study showed that significant differences occurred in the PCNL practice patterns between trained and untrained urologists in Latin America.

In a survey completed by Endourological Society members, 62% of respondents reported that they had received endourology training (5). Latin American urologists have a similar percentage of endourology training, with 66.7% of our respondents reporting that they had received endourology training.

As mentioned above, one of the most important influencing factors on surgical outcomes is a surgeon's case volume, and our results showed that Latin American urologists with training in en-dourology performed a significantly larger number of PCNL procedures than urologists without training. Thus, a proportional relationship was observed, with improved surgical outcomes observed for urologists with a greater case volume of PCNL procedures per year.

The American Urological Association and the European Urological Association have established that PCNL is the gold standard for the treatment of renal stones >2cm because the PCNL procedure has a better stone-free rate than other minimally invasive treatments. However, recent improvements in flexible ureteroscopes have led to the preference of flexible ureteroscopy for the treatment of renal stones of 2-3cm among a number of urologists at high-volume centers (9, 10).

In a study from the United Kingdom, 29% of the PCNL procedures were performed for renal stones >2cm; 33% of the PCNL procedures were performed for renal stones at 1-2cm; and 9% of the PCNL procedures were for stones <1cm (11). These findings show that a greater percentage of PCNL procedures in the United Kingdom corresponded to stones with diameters from 1-2cm. However, our data showed that a greater number of Latin American urologists with training in endourology preferred performing PCNL for renal stones >2cm compared with nontrained urologists (72.9% vs. 51.9%) (Table-4).

**Table 4:** PCNL by indicator among urologists with and without endourology training.

Indication	Endourology training	Without endourology training	p-value*
Renal calculi >20mm at any location	161 (72.9%)	55 (51.9%)	0.0005
Calculi >15mm at any location	60 (27.1%)	12 (11.3%)	0.003
Calculi <20mm in lower pole calyx	77 (34.8%)	24 (22.6%)	0.033
Multiple renal calculi	106 (40%)	31 (29.2%)	0.001
Horseshoe kidney	74 (33.5%)	28 (23.6%)	0.043

* Fisher's exact test was used. Differences were statistically significant at p-values <0.05.

Computed tomography (CT) is the cornerstone for PCNL surgical planning, and AUA guidelines state that CT should be performed for all patients prior to PCNL. Our study showed that CT is more frequently performed before surgery by urologists with endourological training than by urologists without training, and the differences were statistically significant. This pattern could indicate that trained urologists perform more careful surgical planning than those without training, which could represent an additional factor that could influence the final surgical outcomes.

Regarding surgical planning and patient counseling, previous studies have not evaluated the rate at which nephrolithometry scores are used by urologists. These scores are useful for predicting stone-free and transfusion rates as well as the likelihood of complications after PCNL (12–15). However, although the use of nephrolithometry scores is not common among Latin American urologists, urologists with endourological training use these scores significantly more often than urologists without training (12.7 vs. 4.7%).

Trauma to renal parenchyma and bleeding are associated with the tract size. To minimize tract-associated morbidity in PCNL, various urologists worldwide have applied the minimally invasive PCNL procedure (miniperc) followed by the micro-PCNL (microperc) and the ultramini PCNL (UMP) procedures (16–18).

Miniperc defined as a percutaneous tract diameter between 15-20Fr (19) has been used to treat medium-sized (10-20mm) renal stones in 11.7% of patients in high-volume centers (20); thus, it has become popular among urologists. Our data showed that nontrained Latin American urologists were less likely to perform miniperc procedures than trained urologists (5.7% vs. 19.9%, p=0.0005). The purpose of this study was not to investigate the preference of other treatment options for stones <20mm among urologists, such as extracorporeal shock wave lithotripsy, fURS, miniperc, ultraminiperc and microperc. In this study, we investigated only the miniperc preferences for renal stones among the surveyed urologists; therefore, further investigations are required to determine the preferences of urologists for the endourological treatments for stones <20mm.

ECIRS was developed to minimize multiple percutaneous tracts (21); however, our results showed that the percentage of ECIRS procedures and the number of multiple percutaneous tracts was higher in trained urologists than in those who were not trained. A possible explanation for this finding could be that trained urologists performed more PCNL procedures annually than untrained urologists; thus, trained urologists treat more complex cases. Another explanation for this finding could be related to the greater experience and self-confidence of high-volume surgeons.

Tubeless and totally tubeless drainage options are recent modifications of PCNL, and a recent meta-analysis showed that tubeless PCNL has potential advantages, including reduced postop-erative pain and analgesia requirements, shorter hospitalizations and convalescence periods, and lower costs (22). However, Sivalingam and coworkers reported that 76% of urologists (partici-pants were members of the Endourological Society) continued to place a nephrostomy tube at the end of the procedure (5), and Armitage reported that 53% of urologists in the United Kingdom continued to place a nephrostomy tube at the end of the PCNL procedure.

Thus, it appears that urologists worldwide favor the placement of a nephostomy tube for post-operative drainage, which continues to be the predominant procedure upon completion of PCNL. In Latin America, our results showed that nephrostomy tube drainage was the most common postoperative practice pattern; however, our comparison of trained and untrained urologists showed that nephrostomy tube co-location was significantly reduced and ureteral catheter placement (tubeless) was preferred by the trained urologists (Table-3).

The totally tubeless drainage option has a low acceptance rate among urologists, and Armitage re-ported that only 14% of urologists in the United Kingdom do not place a tube after the procedure (including a ureteral catheter) (11). In contrast, totally tubeless drainage (no nephrostomy and no ureteral catheter) has a high preference rate by trained urologists in Latin America; however, significant differences were not observed in the preference rate compared with untrained urologists (Table-3).

CT represents the gold standard imaging procedure for the detection of upper urinary tract stones, and the sensitivity and specificity have been reported to exceed 95% and 99%, respectively. Thus, CT is the ideal scan for evaluating the stone-free rate at the end of any endourological procedure for urinary stone treatment, including PCNL (23).

Sountoulides and colleagues observed that routine follow-up with unenhanced CT is beneficial for patients and complete eradication of stones is essential because of a higher risk of recurrent stone formation (23). However, trained urologists in Latin America show a reduced preference for the use of CT for stone-free rate evaluations.

Determining how training in endourology could affect the practice patterns among urologists dedicated to stone treatment is important, and such training could explain the difference in final surgical outcomes and perioperative and postoperative complications. Our results clearly show that the practice patterns between trained and nontrained urologists differ; however, follow-up studies are necessary to determine the factors that could explain the differences in these practice patterns.

Because our data were obtained via an electronic survey completed by urologists, the findings cannot be used to reflect the exact practice patterns of urologists or establish precise explanations of the observed trends (like the equipment and the access to new technology that each urologist could have); however, the results have some merit. Although our response rate was low (16.55%), it is similar to previous studies reporting a response rate of 14-20% (5, 24–25).

A strength of our study is that it is the first to evaluate the practice patterns of PCNL among urologists in Latin America with and without en-dourology training.

## CONCLUSIONS

Significant differences were observed in the PCNL practice patterns between Latin American urologists with and without endourology training. The preoperative use of nephrolitometric scales and tomography, minimally invasive PCNL, and combined management (ECIRS) and a greater percentage of multiple percutaneous tracts are more commonly observed with trained urologists. Finally, trained urologists have a greater case volume per year than nontrained urologists. Endourology training appears to influence the practice patterns of Latin American urologists when performing PCNL procedures and should be encouraged.

## Appendix

Survey: Percutaneous Nephrolithotomy in Latin America

1. What is your age?
  <30
  30-40
  41-50
  51-60
  >60
2. In which country is your current urology practice?
3. In what state or province do you currently work?
4. Do you have any training or a fellowship in endourology/percutaneous renal surgery or lithiasis?
  Yes
  No
5. Do you perform percutaneous renal surgery for the treatment of kidney stones?
  Yes
  No
6. How many percutaneous nephrolithotomies do you perform per year?
  1-10
  11-30
  31-60
  61-100
  >100
7. Is percutaneous renal surgery your preferred treatment for certain types of renal lithiasis?
  Yes
  No
8. In what specific cases do you consider percutaneous renal surgery as the first-line treatment choice for kidney stones? Indicate all cases.
  Calculus greater than 20 mm in any renal localization
  Calculus greater than 15 mm in any renal localization
  Calculus less than 20 mm in the lower calyx
  Multiple renal stones
  Calculus in horseshoe kidney
  Other (please specify)
9. Do you routinely perform a computerized tomography scan of your patients to plan the surgery?
  Yes
  No
10. Do you use any pre-surgical nomograms or scores to predict the free-lithium status?
  Yes (specify which)
  No
11. Which of the following do you most commonly use to perform percutaneous puncture in the treatment of kidney stones?
  Ultrasound
  Fluoroscopy
  Tomography
  Other (please specify)
12. In fluoroscopic puncture, what technique do you use?
  Eye of the needle
  0-90 degrees
  0-30 degrees
  Other (please specify)
13. What is the average time of fluoroscopy application during percutaneous nephrolithotomy?
14. If necessary, do you perform more than one percutaneous tract in the treatment of renal stones?
  Yes
  No
15. In what position do you prefer to place the patient to perform percutaneous nephrolithotomy for renal stone treatment?
  Prone
  Supine (Valdivia)
  Supine (Valdivia - Galdakao)
  Other (please specify)
16. Do you perform endoscopic combined intrarenal (ureteroscopy+nephrostomy) retrograde surgery for renal stone management?
  Yes
  No
17. In what position do you perform endoscopic combined intrarenal surgery (ureteroscopy+percutaneous nephrolithotomy)?
  Prone
  Supine (Valdivia)
  Supine (Valdivia - Galdakao)
  Other (please specify)
18. What anesthetic method do you prefer when performing percutaneous nephrolithotomy for the treatment of kidney stones?
  Spinal epidural
  Subarachnoid spinal
  Local with sedation
  Local without sedation
  General inhaled
  Other (please specify)
19. Do you consider it useful to perform a culture of the percutaneous puncture urine?
  Yes
  No
20. For the percutaneous tract, what method of dilatation do you commonly use?
  Progressive with Amplatz
  Progressive with Alken
  Dilating balloon
  One shot (Amplatz)
  Other (please specify)
21. For the percutaneous tract, what French size do you prefer when performing dilatation?
  <22 fr
  22 fr
  24 fr
  26 fr
  28 fr
  30 fr
  Other (please specify)
22. Do you usually perform miniperc surgery?
  Yes
  No
23. What instrument size in Fr. units do you use to create the tract in percutaneous mini renal surgery?
  12 fr
  14 fr
  16 fr
  18 fr
  20 fr
24. In which cases do you prefer to perform miniperc surgery?
  Lithos of 15-20 mm
  Less than 15 mm but greater than 10 mm
  Calcium hydroxide
  Limestones less than 10 mm in the inferior calyx that failed to respond to flexible ureteroscopy
  When performing a second percutaneous tract
  Lithos greater than 20 mm
  Other (please specify)
25. For the fragmentation of the renal calculus, what type of energy do you prefer?
  Pneumatic
  Ultrasonic
  Ultrasonic/Pneumatic
  LASER
  Other (please specify)
26. At the end of percutaneous nephrolithotomy, do you
  Place a nephrostomy catheter
  Place a nephrostomy catheter and double ”J” ureteral catheter
  Place double ureteral catheter ”J”
  Do not place a nephrostomy catheter or catheter
  Other (please specify)
27. Which radiological method do you prefer for evaluating the stone-free status?
  Tomography
  Ultrasound
  Simple abdomen plate
  Simple abdomen plate + ultrasound
  Other (please specify)
